# A tutorial for calculating field-specific effect size distributions

**DOI:** 10.3758/s13428-026-03003-2

**Published:** 2026-04-29

**Authors:** Bernt D. Glaser, Heemin Kang, Kristin Audunsdottir, Alina I. Sartorius, Daniel S. Quintana

**Affiliations:** 1 Department of Psychology, University of Oslo, Oslo, Norway; 2 PsychGen Centre for Genetic Epidemiology and Mental Health, Norwegian Institute of Public Health, Oslo, Norway; 3 K.G. Jebsen Centre for Neurodevelopmental Disorders, University of Oslo, Oslo, Norway; 4 Department of Rare Diseases, Oslo University Hospital, Oslo, Norway

**Keywords:** R, Effect size, Effect size distribution, Study design, Power analysis

## Abstract

Effect sizes are useful for understanding the magnitude of study results and for planning new studies via power analysis. However, despite their wide usage, effect sizes are often misinterpreted. This is mostly due to an over-reliance on general effect size benchmarks that were not intended for broad application across diverse research fields. Inaccurate effect size interpretations can lead to incorrect conclusions about the magnitude of study results and incorrect sample size estimates, thereby increasing the likelihood of false-positive results. This article introduces the *ESDist* R package, which is designed to calculate empirically derived effect-size benchmarks or a range of reliably detectable empirical effect sizes for a specific research question or field of interest by computing effect size distributions (ESDs). This package can be used on data that can be easily extracted from pre-existing meta-analyses to help researchers more accurately plan new studies or to better understand how an individual study might relate to other studies in their field. *ESDist* includes a set of features that make it easy to use in a priori power analysis. Moreover, the package includes a feature for estimating effect size benchmarks that account for publication bias and are weighted by effect sizes' variances, which addresses existing limitations of using ESDs for study planning or interpretation.

**Supplementary Information:**

The online version contains supplementary material available at 10.3758/s13428-026-03003-2.

## Introduction

Effect sizes are important empirical study outcomes (Lakens, 2013) which, unlike *p* values, can describe the *magnitude* of an effect, rather than only indicating the *presence* of an effect. This means that effect sizes can be used to make inferences about the practical importance of an observed effect (Cohen, 1988). Unlike an *unstandardized* effect size, which describes an effect size on the same scale as the effect was detected (e.g., weight in kilograms), *standardized* effect sizes (e.g., Cohen’s *d*) describe the magnitude of an association or difference between groups in a way that does not refer to the original measurement scale. As such, standardized effect sizes can facilitate comparisons between studies, regardless of differences in study design, population sample, or methods.

Despite the benefits of standardized effect sizes, their magnitudes can be difficult to interpret because they do not reflect the original measurement scale. The use of Cohen’s (1988) heuristics for small, medium, and large effect sizes for various standardized effect sizes (e.g., Cohen’s *d*, Pearson’s *r*) has become ubiquitous in the psychological and biomedical sciences for both interpreting study results and planning new studies via a priori power analysis, but this was never Cohen’s intention (Funder & Ozer, 2019), as he suggested these heuristics as a last resort for a priori power analysis (Cohen, 1988). That is, researchers should use these heuristics only when effect size estimates from previous research are unavailable. These heuristics have no empirical basis, and therefore do not describe effect sizes as small, medium, or large compared to anything in particular (Funder & Ozer, 2019). The use of imprecise heuristics can lead to issues with understanding the magnitude of individual study results, as well as problems with planning appropriately powered studies (Funder & Ozer, 2019).

The aim of this article is to introduce the *ESDist* package for R (R Core Team, 2023) as an empirical approach for calculating effect size benchmarks for use in a priori power analysis or for the interpretation of observed effects. We will begin by discussing the limitations of using Cohen’s heuristics to interpret study results or to plan new studies. Subsequently, we will argue that the use of field-specific effect size distributions (ESDs) could be a useful alternative to the use of Cohen’s benchmarks. We then discuss how publication bias can influence the calculation of ESDs, consequently inflating effect size benchmarks. Finally, the *ESDist* package for R will be introduced. We demonstrate how *ESDist* can be used to visualize ESDs through various approaches. We also show how to calculate field-specific effect size benchmarks that account for publication bias.

### Issues with general effect size heuristics

**Fig. 1:**
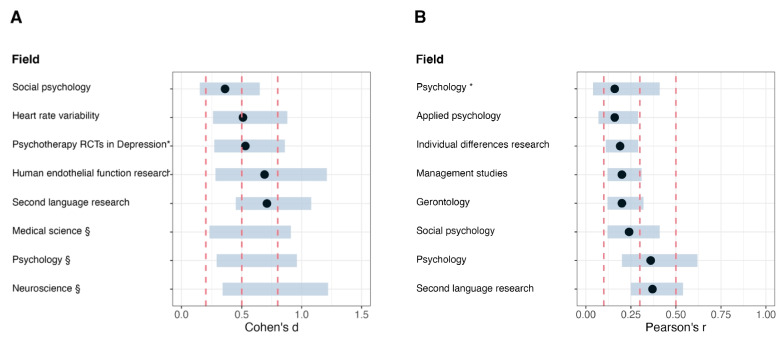
Effect size benchmarks for several fields in the biomedical sciences compared to Cohen’s suggested benchmarks. *Note.* **A** Effect size benchmarks for several fields in Cohen’s *d*. Each *bar* represents an interquartile range (i.e., all effect sizes between the 25th to 75th percentiles). Each dot represents the median, if reported. The *red dashed lines* represent Cohen’s (1988) heuristics for small, medium, and large effect size, which are *d* = 0.2, 0.5, and 0.8, respectively. **B** Effect size benchmarks for several fields in Pearson’s *r*. Each *bar* represents an interquartile range (i.e., all effect sizes between the 25th to 75th percentiles), except the bars for the field of psychology (taken from Schäfer and Schwarz, 2019), where the *bars* represent the range from the 16.65th to the 83.35th percentiles. Each *dot* represents the median. The *red dashed lines* represent Cohen’s (1988) heuristics for small, medium, and large effect size, which are *r* = 0.1, 0.3, and 0.5, Studies with an asterisk (*) have been adjusted for publication bias. Studies with a section sign (§) did not report a median value.

The use of Cohen’s heuristic benchmarks for interpreting results and planning new studies is common across many research fields. A “medium” effect size is supposed to approximate the average effect size in a research field (or for a research question), with “small” and “large” effects equidistant from a medium-sized effect. While Cohen’s benchmarks may happen to align with the distribution of effects for some fields, they cannot be accurate for *all* research fields, as variation in methods, measures, and populations can affect ESDs. Consequently, some researchers have developed field-specific effect size benchmarks from effect size distributions (typically based on the 25th, 50th, and 75th percentiles), which suggest that there is considerable variability between fields when it comes to effect size benchmarks. For example, Gignac and Szodorai (2016) suggested Pearson’s *r* values of 0.11, 0.19, and 0.29 to correspond to relatively small, typical, and relatively large effect sizes, respectively, in the individual differences (i.e., trait psychology) literature. Lovakov and Agadullina (2021) suggested Pearson’s *r* values of 0.12, 0.24, and 0.41, and Cohen’s *d* values of 0.15, 0.36, and 0.65 to correspond to small, medium, and large effect sizes, respectively, in the social psychology literature*.* An overview of some existing effect size benchmarks is presented in Fig. 1A and B. Figure 1A includes six studies that report effect size benchmarks using Cohen’s *d* (Cherubini & MacDonald, 2021; Lovakov & Agadullina, 2021; Nordahl-Hansen et al., 2022; Plonsky & Oswald, 2014; Quintana, 2017; Szucs & Ioannidis, 2017). Figure 1B includes seven studies that report effect size benchmarks using Pearson’s *r* (Bosco et al., 2015; Brydges, 2019; Gignac & Szodorai, 2016; Lovakov & Agadullina, 2021; Paterson et al., 2016; Plonsky & Oswald, 2014; Schäfer & Schwarz, 2019). The corresponding tables can be found in the Supplementary Materials (Supplementary Tables S1 and S2).

### Implications for using general effect size benchmarks for study interpretation and planning

Given this variability of effect size benchmarks across different fields, the use of Cohen’s general effect size heuristics can lead to potential misinterpretations of observed effect sizes, and, in a priori power analysis, this can lead to study test and design combinations that are statistically underpowered to reliably answer specific research questions (Lakens, 2022). Statistical power, the probability of correctly rejecting the null hypothesis if there is a true effect, is a function of study design, hypothesized effect size, sample size, and alpha level. Statistical power increases as either the sample size or the hypothesized effect size increases. When designing a study, researchers should determine a sample size that provides sufficient statistical power, typically 80%, although some researchers suggest 90–95% instead (e.g., Lakens, 2022). Inaccurate justification of the effect size parameter in a priori power analysis can lead to studies that cannot reliably detect a wide range of plausible effect sizes (Brydges, 2019). For example, if a researcher expects to find a ‘medium-sized’ effect, they might plan for a one-tailed paired-samples *t* test using Cohen’s suggestion for a medium effect size (*d* = 0.5) with 80% power, which would require a sample size of *N* = 27. However, if the ‘true’ medium effect size (i.e., the 50th percentile effect size) for their specific field is *d* = 0.4, the study would only have 64.8% power, meaning that there would be a considerable chance of not detecting an effect even though it exists.

There are several ways in which effect sizes can be selected more accurately when performing an a priori power analysis. Cohen (1988) originally proposed that the effect size parameter in a priori power analysis should be treated as an expected population effect size. However, since the population effect size is almost always unknown, authors have more recently advocated that researchers should treat this parameter as the smallest effect size of interest (SESOI) instead (Lakens & Evers, 2014). With this approach, the researcher decides on the smallest effect size that is deemed ‘worthwhile’ and designs a study that is adequately powered to detect this effect size. Consequently, the power level is sufficiently high to detect this smallest effect size of interest, and power only increases for larger effect sizes. A different, yet related, approach is sensitivity power analysis, where the researcher uses a given sample size as a starting point instead of an effect size of interest (Lakens, 2022). This approach is useful when data is already collected, or there is a known limit to the number of participants that can be tested, for example due to resource constraints. In this case, the goal is to gain insight into what *range* of effect sizes can be detected for a range of acceptable power levels. Consequently, the aim is to strike a balance between the effect sizes that are meaningful to detect or reject and the level of accuracy the researcher wishes to achieve in detecting or rejecting these effects. However, even with these more sensible approaches to power, researchers need to have a good understanding of which effect sizes are interesting for their research question to justify the effect size parameter in their power analysis (Lakens, 2022). Alternatively, researchers can use summary effect sizes from existing meta-analysis as a proxy for an “average” effect. However, if the summary effect size was not adjusted for publication bias, it could be inflated from the “true” effect. Moreover, this approach can only be used for approximating medium effects, not small or large effects.

### The calculation of field-specific benchmarks using ESDs

Although field-specific benchmarks like those presented in Fig. 1A and B are likely to outperform Cohen’s more general heuristics, many fields for which these heuristics have been developed are still very broad (e.g., “psychology” or “medical science”). Moreover, such benchmarks are not widely available, especially for more specific fields. In many cases, researchers would benefit from having access to an ESD that is specific to their field, sub-field, or even to a particular research question. A field-specific ESD would allow researchers to better understand the magnitude of their observed effect compared to previous research within the field, either by calculating field-specific benchmarks or by calculating the percentile that corresponds to their observed effect size.

Having access to an ESD might be even more useful when planning new studies. For example, researchers might have specific reasons for setting their SESOI to correspond to a particular percentile in their ESD, such as the quartiles conventionally associated with small, medium, or large effect size benchmarks. Alternatively, if a researcher knows their sample size parameter, they can perform a sensitivity power analysis, which creates a range of reliably detectable effect sizes. Subsequently, they can use an ESD to calculate the proportion of empirical effect sizes in the ESD that fall within this range for each level of power. Knowing the range of empirical effect sizes that can be reliably detected given a particular study design is useful in several cases. For example, if the SESOI is so high that the percentage of effect sizes that can be detected by the study is close to 0%, it is unlikely that the study will provide any new information to the field (e.g., Lakens et al., 2018).

### Existing limitations of field-specific ESDs

Because field-specific ESDs largely consist of effect sizes reported in published studies, it is likely that they are influenced by publication bias (Button et al., 2013; Lakens, 2022). Publication bias (also known as ‘non-reporting bias’; Page et al., 2024) refers to the phenomenon where studies with statistically significant results are more likely to be published than studies without a significant result, which, in turn, leads to an overall inflation of effect sizes (Kvarven et al., 2020; Schäfer & Schwarz, 2019). This is an issue that mainly affects small studies (Dwan et al., 2008). Non-significant effects in small studies are often attributed to low power and are therefore deemed inconclusive or uninformative (Evangelou et al., 2012). The publication of small sample studies with non-significant results is decided against (either by journal editorial teams or the authors not submitting the study for publication in the first place) more frequently than large studies with non-significant results, since non-significant results in studies with a large sample size are more difficult to explain on the basis of low power (Button et al., 2013). Meanwhile, small studies that do find effects are more likely to be published, despite the higher likelihood that they greatly overestimate the true effect size (Rochefort-Maranda, 2021). Aside from publication bias, small studies are more sensitive to other biases, such as vibration effects (i.e., the variability in a study’s effect size estimate based on the selection of analysis method), selective data analysis and selective reporting, and reduced quality of other aspects of the study design (Button et al., 2013). Given these various biases, any estimate of the population effect size that is based on previous literature is likely to be an overestimation in most cases (Lakens, 2022). Therefore, it is essential to address these biases when interpreting individual study results or planning a new study using an ESD.

Another potential issue with ESDs is that effect sizes are not equally informative. The effect size of each study is merely an estimate of the “true” effect size. As such, each effect size also has a standard error, which is a measure of the precision of the effect size estimate. ESDs are not currently calculated with a consideration of effect size precision. This means that effect sizes influence an ESD calculation equally, regardless of their precision. Because more precise estimates are more informative than less precise ones, it may be desirable to assign greater weight to them. While the weighting of effect sizes is commonplace in meta-analysis via inverse variance weighting, this is not a consideration when ESDs are typically calculated.

Finally, the existing practice of using a field-specific ESD to calculate benchmarks for small, medium, and large effects does not involve an assessment of the *precision* of such estimates, to our knowledge, such as standard errors or 95% confidence intervals (CIs). Given that the calculation of benchmark estimates is often based on somewhat arbitrary percentiles (most commonly quartiles), these estimates can range in precision depending on the skewness of the ESD, outliers, the distance of the percentile from the mean, and the number of effect sizes available in the field. When 95% CIs for benchmark estimates overlap, this could indicate that the field of interest is not sufficiently mature to establish precise effect size benchmarks.

## The *ESDist* package for R

Although ESDs are useful for interpreting effect sizes and for planning studies, they are not widely available. One reason for the low availability of ESDs might be the amount of effort required for gathering effect size estimates of individual studies within a specific field of interest. Moreover, even if many effect size estimates are readily available, researchers might find it difficult or time-consuming to gather all relevant information from their ESD, such as effect size benchmarks, the range of reliably detectable effect sizes, or the weighted distribution of empirical effect sizes. Furthermore, even if researchers can obtain field-specific ESDs, publication bias in the ESD might render any information gained from the ESD inaccurate.

To this end, the aim of this paper is to introduce the *ESDist* package for the R statistical software suite (R Core Team, 2023). The *ESDist* package was designed to facilitate the calculation and visualization of field-specific ESDs based on data that can easily be retrieved from meta-analyses, for the purpose of interpreting individual effect sizes and planning new studies. Moreover, by combining the *ESDist* package with the *meta* (Balduzzi et al., 2019) and *metasens* (Schwarzer et al., 2015) packages, users can justify their SESOI based on an ESD that is adjusted for publication bias. In the following sections, we will explore the *ESDist* package by demonstrating its functions, accompanied by their respective outputs. Moreover, reasons for why the use of the *ESDist* package may be preferential over other benchmark-based methods, as well as potential limitations and areas of future investigation and development, will be discussed.

### Package overview

**Fig. 2:**
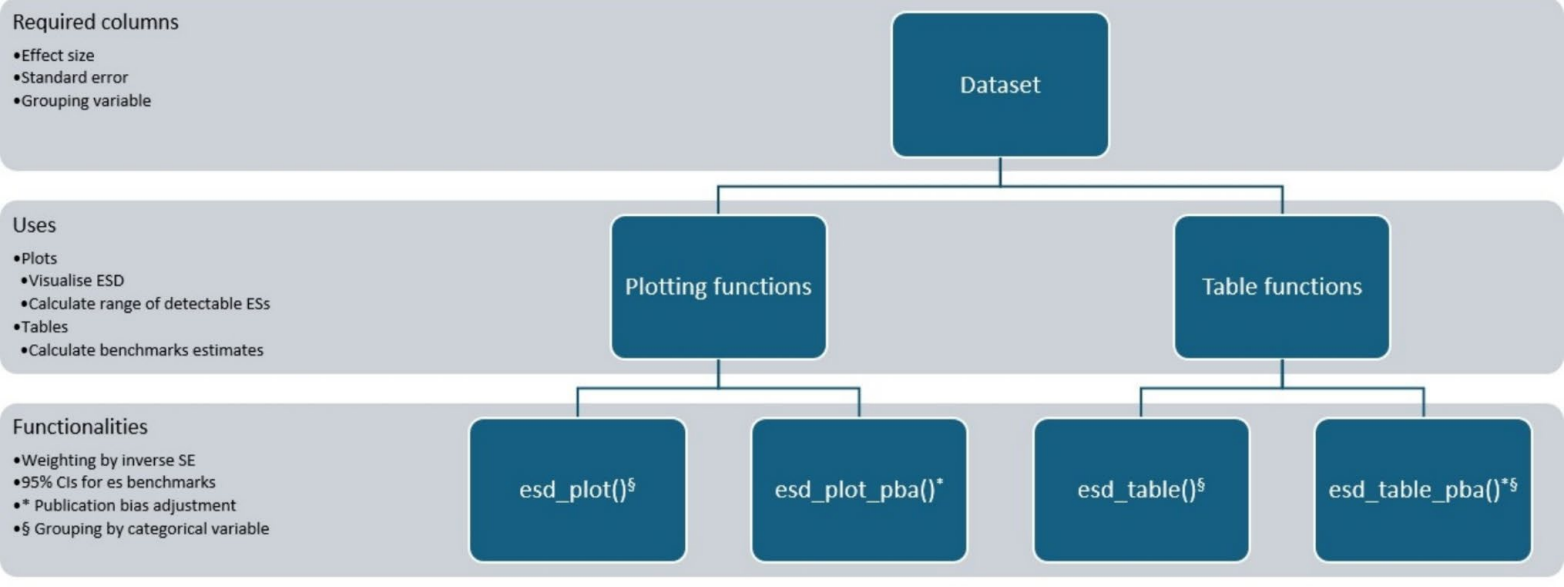
An overview of all main functions in the *ESDist* package. The dataset that is to be used with the *ESDist* package should contain at least columns for effect size, standard error, and a grouping variable to make use of all functions in the package. The functions in the package can be divided into two categories: plotting functions and table functions. The plotting functions are primarily useful for visualizing ESDs, as well as for calculating the range of detectable empirical effect sizes, whereas table functions are primarily useful for calculating estimates for effect size benchmarks. Finally, all functions allow for inverse weighting by standard error to make more precise estimates in the dataset more informative. Functions with an *asterisk* (*) also allow for publication bias adjustment, and functions with a section sign (§) allow for grouping by a categorical variable

The *ESDist* package contains functions for visualizing ESDs, as well as a function for calculating effect size benchmarks. The *ESDist* package requires R version 2.10 or higher and can be loaded using the following commands:
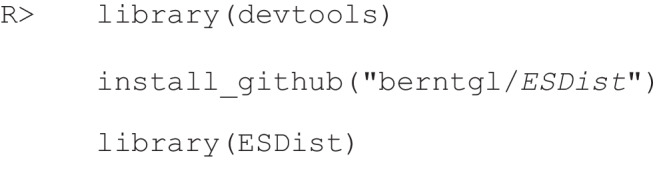


These commands first load the *devtools* package (Wickham et al., 2022), which is necessary to download and install packages from GitHub. The *ESDist* package is then downloaded from GitHub and loaded into the user’s R environment. An overview of all uses and functionalities of each main function can be found in Fig. 2.

### Data files

The package contains two example data files: *ot_dat_raw* and *ot_dat*. The data file *ot_dat_raw* contains 616 effect sizes from 15 meta-analyses on oxytocin intervention studies (Bakermans-Kranenburg & van IJzendoorn, 2013; Chen et al., 2021; Huang et al., 2021; Kang et al., 2022; Keech et al., 2018; Leppanen et al., 2017, 2018; Leslie et al., 2018; Ooi et al., 2017; Peled-Avron et al., 2020; Sabe et al., 2021; Wang et al., 2019; Yang et al., 2021; Zheng et al., 2019; Zhou et al., 2021). Oxytocin is a hormone and neuromodulator that is primarily produced in the hypothalamus (Jurek & Neumann, 2018). It has been the subject of comprehensive research interest due to its role in social behavior, cognitive regulation, and metabolic homeostasis (Jurek & Neumann, 2018; Quintana & Guastella, 2020).

Some studies were included multiple times in the dataset because they reported multiple effect sizes. Thus, the package contains a second, processed data file, *ot_dat*, where duplicates have been removed, and all effect sizes have been converted to the same metric (Hedges’ *g*). When a study was included multiple times in the original dataset, only the effect size with the smallest standard error was considered. In the unlikely case that the standard error was the same between two effect sizes of the same study, only the smallest effect size (i.e., the one closest to 0, regardless of the direction) was considered. As a result, the updated dataset contains 182 effect sizes. Both datasets contain a column with effect sizes, *yi*. All effect sizes in this column are reported as Hedges’ *g* in the *ot_dat* dataframe, or as the original effect size in the *ot_dat_raw* dataframe. The dataset also contains a column, *sei*, that contains the standard error of the effect sizes. The dataset includes information on each study, such as sample size (*n_total*), study year (*study_year*), study design (*design*), oxytocin dosage (*doses*), the clinical diagnosis of the subsample (*group*), and details about the meta-analysis from which the study originated (*meta_analysis*, *meta_analysis_doi*, *meta_analysis_year*). Users can get an overview of all the included columns and the data they contain by using the following command:
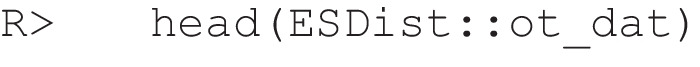


### Analysis script and data filtering script

To aid users in using the *ESDist* package, an example analysis script (*analysis_script.R*) is included in the package, which contains examples of all functions and their features. This script, as well as a script (*data_filtering.R*) for processing the raw data (*ot_dat_raw*) to obtain the filtered dataset (*ot_dat*) are included in the supplementary materials (Supplementary files 1 and 2) and can also be accessed through https://github.com/berntgl/ESDist.

### Calculating effect size benchmarks in tables

The *esd_table()* function is the primary table function in the *ESDist* package and can be used to calculate field-specific effect size benchmarks. In its most basic form, it takes two arguments: a data frame (*df*) and the column within that dataset that contains effect sizes (*es*). By default, the function will calculate the small, medium, and large effect size benchmarks based on the 25th, 50th, and 75th percentiles, respectively. By setting the *method* argument to “thirds”, the function will calculate the 16.65th, 50th, and 83.35th percentiles instead. The code below sets the *df* argument to the *ot_dat* data frame and the *es* argument to the column within that data frame which contains effect sizes, *yi*. The output of this function is presented in Table 1.

**Table 1:** The output of the esd_table() function for all effect sizes in the ot_dat dataset

	25 %	50 %	75 %	Number of effects
Raw effect size	0.06	0.23	0.50	182

These are the benchmarks for small, medium, and large effect sizes based on the *ot_dat* dataset. The benchmarks are based on the 25th, 50th, and 75th percentiles, which correspond to the ‘quads’ method


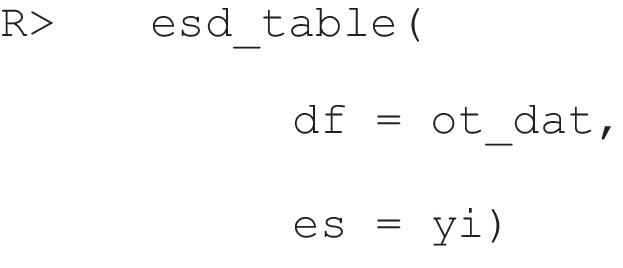


Another feature of the *esd_table()* function is that it can calculate benchmarks per group. The code below specifies that benchmarks should be calculated for each diagnosis group in the *ot_dat* dataset with at least three effect sizes (the minimum number of effect sizes necessary to calculate three benchmarks), by setting *grouping_var* to "group". The resulting table is reported in Table 2. Note that the last row of the table summarizes the benchmarks for all effect sizes included in the dataset.

**Table 2:** The output of the esd_table() function for all effect sizes in the ot_dat dataframe per group

Group	25%	50%	75%	Number of effects
AN	0.02	0.05	0.06	6
Autism	0.11	0.31	0.55	32
BPD	0.16	1.22	2.29	5
PTSD	0.2	0.32	0.38	6
SCZ	0.05	0.13	0.24	20
Anxiety	0.19	0.31	0.41	4
Depression	0.21	0.45	0.91	6
Neurotypical	0.05	0.25	0.66	89
All	0.06	0.23	0.5	182

These are the benchmarks for small, medium, and large effect sizes for each group in the *ot_dat* dataset with at least 4 effect sizes. The benchmarks are based on the 25th, 50th, and 75th percentiles, which correspond to the ‘quads’ method. AN = Anorexia nervosa; BPD = Bipolar disorder; PTSD = Post-traumatic stress disorder; SCZ = Schizophrenia


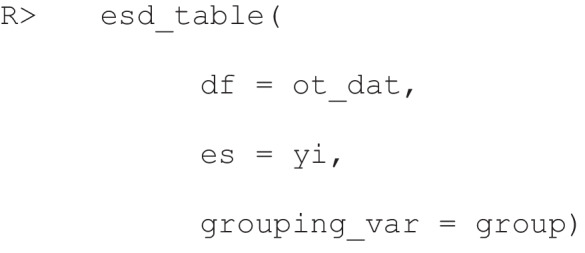


### Basic plotting function

**Fig. 3:**
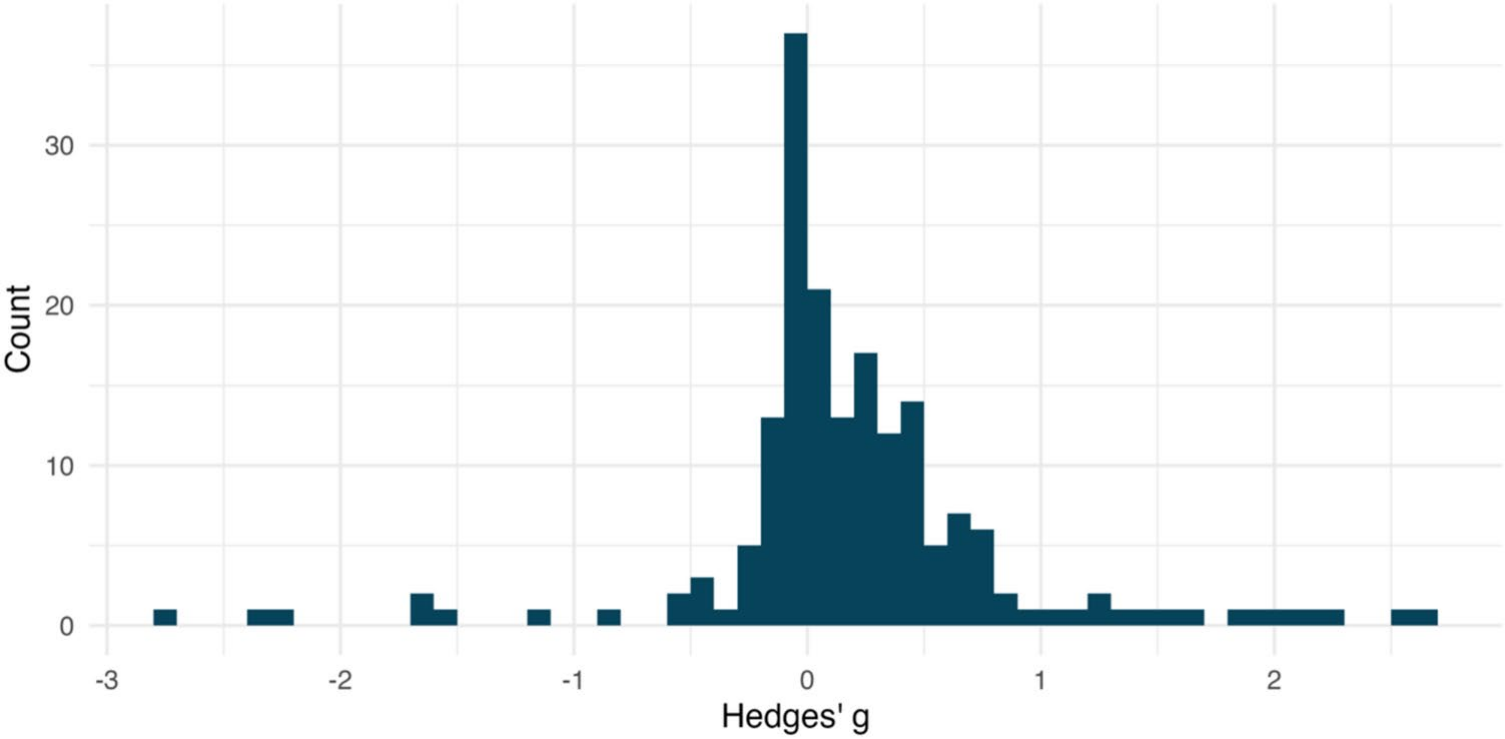
The outcome of the *esd_plot()* function. This histogram shows the distribution of effect sizes for the *ot_dat* dataset. Each bin represents the number of effect sizes within an interval of *g* = 0.1


The most basic plotting function in the *ESDist* package, *esd_plot()*, takes two arguments to create an ESD plot: a data frame (*df*) and the column within that data frame that contains all the effect sizes (*es*). Although not strictly necessary, it is also recommended to provide a string that describes the type of effect size (*es_type*). The *esd_plot()* function takes these arguments and outputs a *ggplot* object. The code below demonstrates the function using the *ot_dat* data frame to generate the plot in Fig. 3. The column in the *ot_dat* dataset that contains all effect sizes is entitled *yi*, and the effect size measure is Hedges’ *g*.

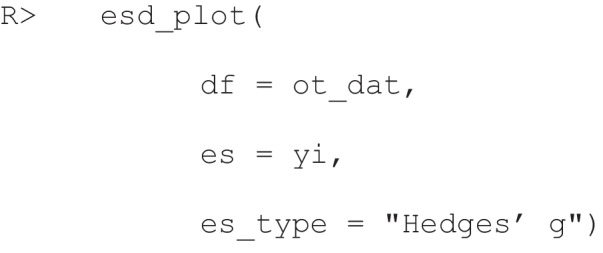


**Fig. 4:**
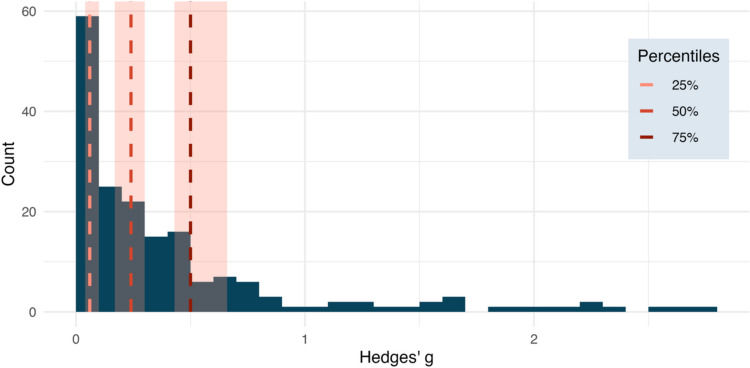
The outcome of the *esd_plot()* function. This histogram shows the absolute distribution of effect sizes for the *ot_dat* dataset, along with vertical lines representing the benchmark estimates for small, medium, and large effect sizes based on the “quads” method (i.e., based on the 25%, 50%, and 75% percentiles). The *transparent red ribbons* around the benchmark estimates represent the 95% CIs. Each bin represents the number of effect sizes within an interval of *g* = 0.1


By setting the *method* argument to *“quads”*, the user can plot the small, medium, and large effect size benchmarks based on the 25th, 50th, and 75th percentiles, respectively (or on the 16.65th, 50th, and 83.35th percentiles if set to *“thirds”*). By additionally setting the *ci* argument to TRUE, the function will plot the 95% CIs around the benchmark estimates (also see “Evaluating robustness via the calculation of skewness and precision”). The code below plots the absolute ESD along with benchmark estimates and their corresponding 95% CIs as shown in Fig. 4.

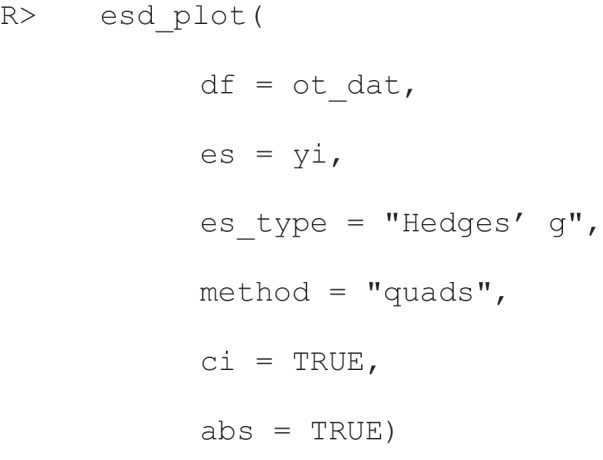


**Fig. 5:**
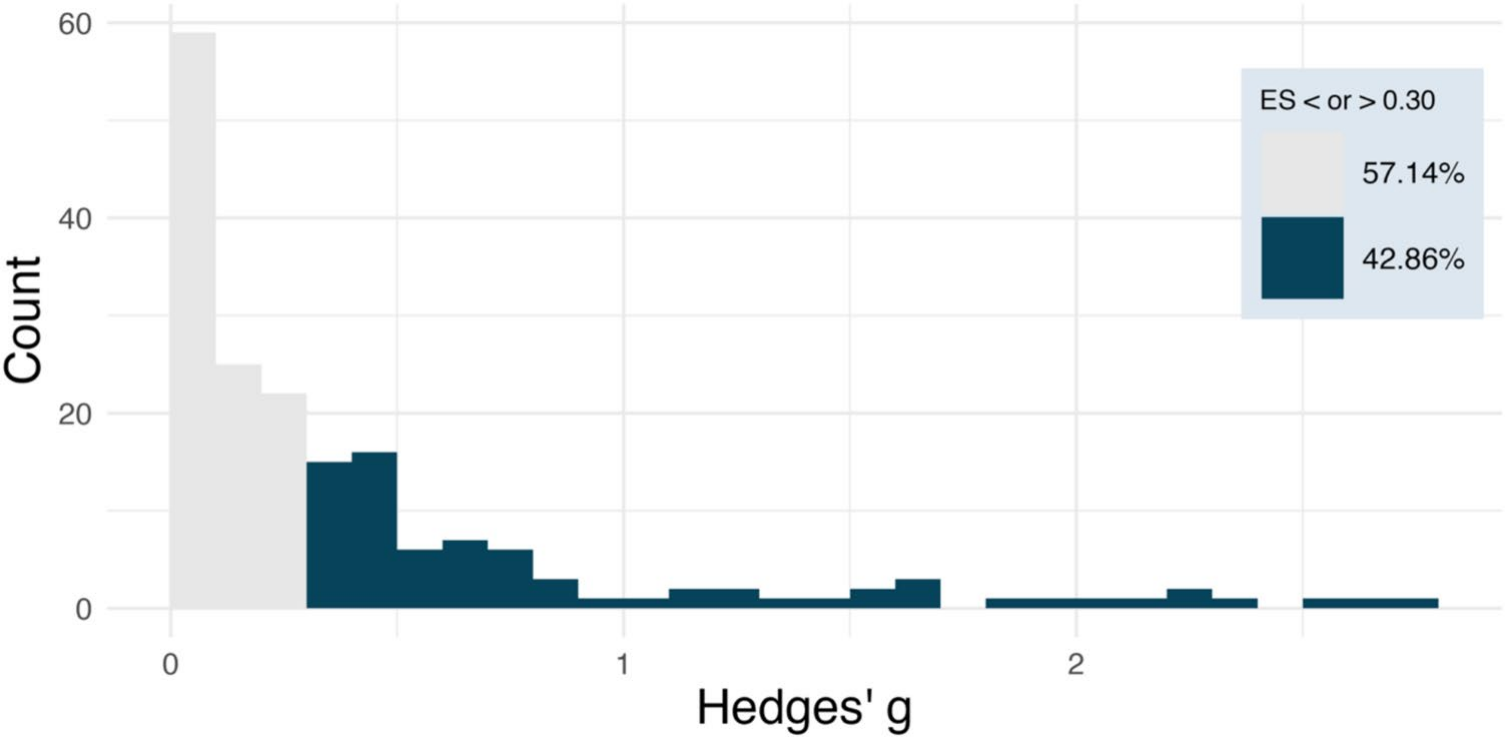
The outcome of the *esd_plot()* function. This plot shows which proportion of the absolute ESD is larger than the given *sesoi* value (0.3) in *dark blue*, and the remaining proportion of the distribution in *grey*


Alternatively, if the user wants to plot the range of empirical effect sizes that fall above or below an effect size of interest, the user can provide the *sesoi* argument with a numerical value. For example, if the user finds that the smallest effect size they can reliably detect with a given level of power is *g* = 0.3, the user may set the *sesoi* argument to 0.3. The returned plot will then visualize what percentage of the absolute ESD is smaller than 0.3, and which percentage is equal to or greater than the given value. This can be done by using the following code, which generates the plot in Fig. 5:

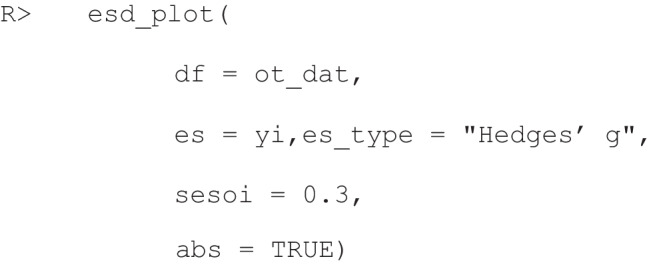


### Publication bias

The *ESDist* package contains two functions, *esd_plot_pba()* and *esd_table_pba()*, that allow users to adjust their ESD for publication bias. These functions make use of the limit meta-analysis method (Rücker et al., 2011; Schwarzer et al., 2015). Limit meta-analysis uses a random-effects model that includes a parameter that accounts for small-study effects for the potential adjustment of individual effect sizes, which makes it a suitable adjustment method for ESDs. The *pba* functions plot and/or calculate effect size benchmarks based on both the original ESD and the publication bias-adjusted ESD.

As with the *esd_table()* function, the *esd_table_pba()* function takes the standard *df* and *es* arguments, but additionally requires the *se* argument, which corresponds to the column of the dataset that contains the standard errors. All options that are available for the *esd_table()* function are also available for the *esd_table_pba()* function. The code below generates data presented in Table 3.

**Table 3:** The output of the esd_table_pba() function for all effect sizes in the ot_dat dataset

	25 %	50 %	75 %	Number of effects
Raw effect size	0.06	0.24	0.50	182
Adjusted effect size	0.08	0.20	0.39	182

These are the benchmarks for small, medium, and large effect sizes for each group in the ot_dat dataset with at least 4 effect sizes, as well as the benchmarks adjusted for publication bias. The benchmarks are based on the 25th, 50th, and 75th percentiles, which corresponds to the ‘quads’ method


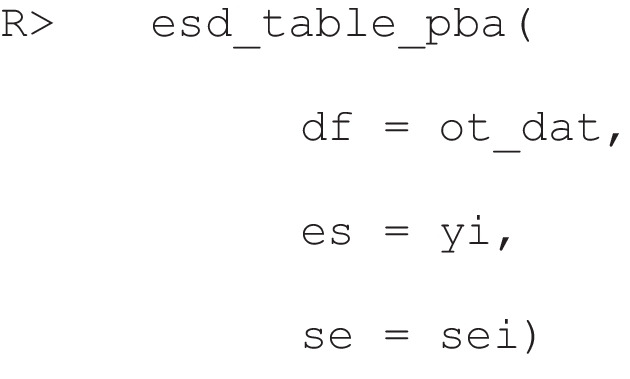


**Fig. 6:**
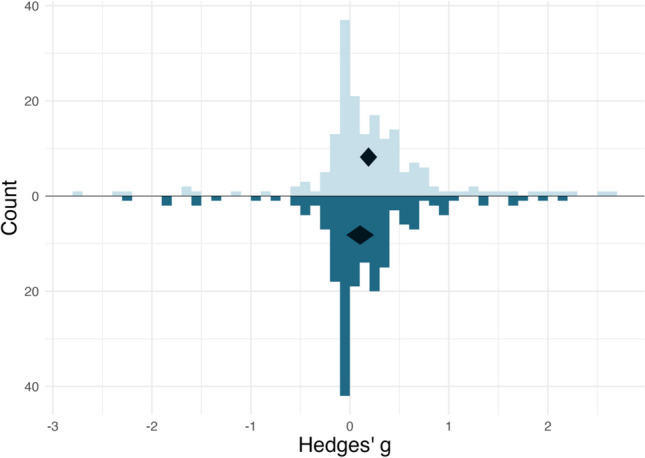
The outcome of the *esd_plot_pba()* function. This iceberg plot shows the distribution of unadjusted effect sizes (top distribution in *light blue*) as well as the distribution of effect sizes adjusted for publication bias according to the limit meta-analysis (bottom distribution in *dark blue*). The summary effect sizes with the 95% CI are shown in the *black diamonds* in each distribution. Each bin represents the number of effect sizes within an interval of *g* = 0.1. CI = confidence interval


The *esd_plot_pba()* function allows users to plot an “iceberg” plot, which shows the unadjusted ESD with the ESD adjusted for publication bias in the negative direction. This function also additionally requires the *se* argument corresponding to the column containing the standard errors. Apart from creating independent plots for groups, the *esd_plot_pba()* function has the same functionalities as the standard *esd_plot()* function. An example of an iceberg plot is shown in Fig. 6, which is generated using the following code:

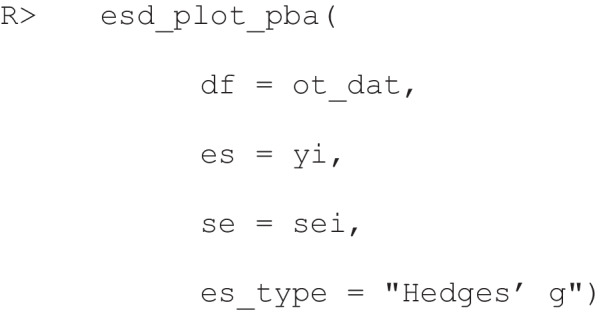


As there is no current gold standard for publication bias adjustment, we echo previous recommendations (Carter et al., 2019; McShane et al., 2016) that researchers use several publication bias adjustment methods as a sensitivity analysis, to compare the summary effect size calculated using limit meta-analysis against other publication bias adjustment methods (i.e., weight selection models and trim-and-fill). We demonstrate how to perform additional publication bias tests in Supplementary File 3.

### Evaluating robustness via the calculation of skewness and precision

To ensure the information about the ESD is as informative as possible, we propose a set of robustness checks to be performed prior to extracting any other information about the ESD. We recommend that users assess the skewness of their ESD to assess whether small or large effects contain a smaller or larger ‘spread’ of effects, as well as whether some form of bias might be present in the distribution (e.g., small study bias might lead to a right-skewed distribution). One measure of skewness built into the *ESDist* package is Bowley’s skewness coefficient (Bowley, 1920). This coefficient is based on the middle 50% of the distribution and ranges from – 1 to 1, where 0 indicates an absence of skewness, and values of – 1 and 1 indicate left- and right skewness, respectively. The table functions within the *ESDist* package allow users to calculate Bowley’s coefficient of skewness or their ESD by setting the *bowley* argument to TRUE.

We also recommend that users evaluate the precision of effect size benchmarks, as the precision of the estimates will likely vary depending on several factors, such as skewness, distance from the mean, outliers, and number of effects in the ESD. As such, the main functions in the *ESDist* package allow users to calculate 95% CIs around the benchmark estimates. CIs are calculated by bootstrapping each benchmark quantile and by subsequently extracting the 2.5% and 97.5% percentiles from the bootstrapped distribution. The *ESDist* package can calculate 95% CIs by setting the *ci* argument to TRUE whenever the *method* argument is provided (which is the default for the table functions, but not the plotting functions). An added benefit of CIs around the effect size benchmarks is that they provide users with a useful heuristic as to whether their ESD contains enough effect sizes. If CIs for small, medium, or large effect sizes overlap, this may indicate the ESD does not contain enough effects to offer precise estimates or that distribution skewness has influenced precision. See “Basic plotting function” for instructions on how to plot these CIs.

### Additional functionalities

The table and plotting functions in the *ESDist* package contain additional functionalities to increase the amount of information that can be drawn from field-specific ESDs. First, since ESDs are often used to gain information on the *magnitude*, and not the *direction* of effects, it is common practice to only use absolute effect sizes. To this end, each function contains an *abs* argument, which plots or calculates benchmark estimates based on absolute effect sizes only. Note that, for the *pba* functions, the publication bias-adjustment is applied prior to converting all effect sizes to absolute values. Second, to address heterogeneity within an ESD, all functions in the *ESDist* package [apart from the  *esd_plot_pba()* function] allow users to plot or calculate effect size benchmarks based on ESDs for each subgroup in their dataset by providing the *grouping_var* argument with the column by which the data should be grouped. Third, if standard errors are provided, all functions allow for each effect size to be weighted by the inverse variance (i.e., 1/SE^2^). Since larger studies often have smaller standard errors, this method gives greater weight to more precise studies, thereby removing some of the imprecision introduced by estimates from smaller studies. To plot a weighted distribution or to calculate effect-size benchmarks based on the weighted distribution, the *se* argument must be set to the column containing the standard errors, and the weighted argument must be set to TRUE.

## Summary

The *ESDist* package for R is a tool that facilitates straightforward calculations of field-specific effect size benchmarks and ESD visualizations. Importantly, the package allows users to create ESDs using data readily available from meta-analyses, and it facilitates adjusting the effect size distribution for publication bias and effect size precision.

There are two primary use cases for the *ESDist* package. The first is to help plan new studies based on information gained from a field-specific ESD. The *ESDist* package allows researchers to calculate field-specific effect size benchmarks based on a set of percentiles (e.g., the 25th, 50th, and 75th percentiles) that correspond to ‘small’, ‘medium’, and ‘large’ effect sizes, respectively. These benchmarks can be used in place of more general effect size benchmarks or heuristics (such as those proposed by Cohen, 1988) to calculate the sample size required to detect a given effect size with a specified level of certainty. Alternatively, researchers can use the *ESDist* package to calculate the range of effect sizes that can be reliably detected with a given study test and design combination. This is particularly useful in situations where there is a known limit on the number of participants who can be tested in a study, as it allows researchers to gauge whether the planned study could make a valuable contribution to its field.

The second use case for the *ESDist* package is for interpreting new study results. For example, researchers can use the *ESDist* package to calculate effect size benchmarks for their specific field of interest to compare new study results against. Alternatively, researchers can avoid using descriptive effect size benchmark labels altogether and report the percentile of the ESD that their study result corresponds to. In other words, instead of making statements such as “we found an effect that was small-to-medium in size”, researchers can state the specific percentile of the ESD that corresponds to the effect size (e.g*.,* “we found an effect that was larger than 34% of effect sizes reported in the field”). Although this is a more precise approach, it should be noted that situating an individual study within an ESD is by no means a complete interpretation of a study result in and of itself (Panzarella et al., 2021; Pogrow, 2019). Researchers should therefore aim to explain their study results within the context of their study design, as well as describe the position of their study within their respective ESD by comparing their study to other studies within the field.

Publication bias is a pressing issue in the psychological sciences, in which some studies are more likely to be published than others (Button et al., 2013; Lakens, 2022). Consequently, effect size estimates drawn from empirical studies may be inflated. The *ESDist* package allows users to account for publication bias by incorporating limit meta-analysis (Rücker et al., 2011; Schwarzer et al., 2015). The *ESDist* package can be used to visualize the adjusted ESD relative to the original ESD via an iceberg plot, or to calculate adjusted effect-size benchmarks. Using a publication bias-adjusted ESD may give the user a more accurate representation of how effect sizes are distributed and will subsequently lead to more accurate estimations of sample size.

The *ESDist* package can be a useful tool for justifying the parameters of a power analysis or for interpreting new study results. Nevertheless, there are some limitations that are worth mentioning. The package was designed for use with data that can easily be obtained from meta-analyses, but there are potential drawbacks to this method. This approach assumes that meta-analyses are available for the field of interest, that these meta-analyses are of sufficient size to create an informative ESD, and that these meta-analyses have reported error-free data. It is recommended that users double-check any unrealistically large values when extracting data from existing meta-analyses.

While ESDs with larger samples are desirable, researchers need to be aware that adding studies with too much heterogeneity (e.g., studies using different methods or populations) can reduce the specificity of the ESD for a particular study. Heterogeneity, in this case, refers to the variety in results obtained from studies that are conceptual or close replications, which makes it difficult to obtain relevant effect size benchmarks or empirical effect size ranges for any particular study (Linden & Hönekopp, 2021). Given that research methods, populations, and measurements might differ substantially between (or even within) scientific fields, there is a good chance that including too many effect sizes could lead to a very heterogeneous pool of empirical effect sizes (Bryan et al., 2021; Higgins & Thompson, 2002; Linden & Hönekopp, 2021). Heterogeneity can be addressed by using the grouping feature included in several functions within the package. It is recommended that users take note of reported heterogeneity of the meta-analyses from which they retrieved their data or perform their own test of heterogeneity (Linden & Hönekopp, 2021).

Altogether, the goal of this tutorial is to instruct readers on generating field-specific effect size distributions to calculate and visualize effect size benchmarks or ranges of detectable empirical effect sizes, aiding researchers in planning new studies with the *ESDist* package. The package can be used with data that can be extracted from pre-existing meta-analyses and can help researchers plan informative new studies a priori and gain information about how an individual study might relate to other studies in their field of interest by using conventional effect size benchmark percentiles for small, medium, or large effects (i.e., the 25th, 50th, and 75th percentiles), or by using more precise percentage values.

## Supplementary Information

Below is the link to the electronic supplementary material.

Supplementary file1 (DOCX 18 KB)

Supplementary file2 (DOCX 15 KB)

Supplementary file3 (DOCX 15 KB)

Supplementary file4 (PDF 104 KB)

## Data Availability

The data can be accessed as a part of the R package code, which is located at https://github.com/berntgl/ESDist.

## Code availability

The code is located at https://github.com/berntgl/ESDist.

## References

[CR1] Bakermans-Kranenburg, M. J., & van IJzendoorn, M. H. (2013). Sniffing around oxytocin: Review and meta-analyses of trials in healthy and clinical groups with implications for pharmacotherapy. *Translational Psychiatry,**3*(5), Article 5. 10.1038/tp.2013.34 10.1038/tp.2013.34 PMC3669921 23695233

[CR2] Balduzzi, S., Rücker, G., & Schwarzer, G. (2019). How to perform a meta-analysis with R: A practical tutorial. *BMJ Mental Health,**22*(4), 153–160. 10.1136/ebmental-2019-300117 10.1136/ebmental-2019-300117 PMC10231495 31563865

[CR3] Bosco, F. A., Aguinis, H., Singh, K., Field, J. G., & Pierce, C. A. (2015). Correlational effect size benchmarks. *Journal of Applied Psychology,**100*(2), 431–449. 10.1037/a0038047 25314367 10.1037/a0038047

[CR4] Bowley, A. L. (1920). *Elements of statistics* (4th ed.). Scribner’s.

[CR5] Bryan, C. J., Tipton, E., & Yeager, D. S. (2021). Behavioural science is unlikely to change the world without a heterogeneity revolution. *Nature Human Behaviour,**5*(8), Article 8. 10.1038/s41562-021-01143-3 10.1038/s41562-021-01143-3 PMC8928154 34294901

[CR6] Brydges, C. R. (2019). Effect size guidelines, sample size calculations, and statistical power in gerontology. *Innovation in Aging,**3*(4), Article igz036. 10.1093/geroni/igz036 31528719 10.1093/geroni/igz036 PMC6736231

[CR7] Button, K. S., Ioannidis, J. P. A., Mokrysz, C., Nosek, B. A., Flint, J., Robinson, E. S. J., & Munafò, M. R. (2013). Power failure: Why small sample size undermines the reliability of neuroscience. *Nature Reviews Neuroscience,**14*(5), Article 5. 10.1038/nrn3475 10.1038/nrn3475 23571845

[CR8] Carter, E. C., Schönbrodt, F. D., Gervais, W. M., & Hilgard, J. (2019). Correcting for bias in psychology: A comparison of meta-analytic methods. *Advances in Methods and Practices in Psychological Science,**2*(2), 115–144. 10.1177/2515245919847196

[CR9] Chen, C.-Y., Chiang, Y.-C., Kuo, T.-C., Tam, K.-W., & Loh, E.-W. (2021). Effects of intranasal oxytocin in food intake and craving: A meta-analysis of clinical trials. *Clinical Nutrition,**40*(10), 5407–5416. 10.1016/j.clnu.2021.08.011 34600216 10.1016/j.clnu.2021.08.011

[CR10] Cherubini, J. M., & MacDonald, M. J. (2021). Statistical inferences using effect sizes in human endothelial function research. *Artery Research,**27*(4), Article 4. 10.1007/s44200-021-00006-6 10.1007/s44200-021-00006-6 PMC8654719 34966462

[CR11] Cohen, J. (1988). *Statistical power analysis for the behavioral sciences* (2nd ed.). L. Erlbaum Associates.

[CR12] Dwan, K., Altman, D. G., Arnaiz, J. A., Bloom, J., Chan, A.-W., Cronin, E., Decullier, E., Easterbrook, P. J., Elm, E. V., Gamble, C., Ghersi, D., Ioannidis, J. P. A., Simes, J., & Williamson, P. R. (2008). Systematic review of the empirical evidence of study publication bias and outcome reporting bias. *PLoS One,**3*(8), Article e3081. 10.1371/journal.pone.0003081 18769481 10.1371/journal.pone.0003081 PMC2518111

[CR13] Evangelou, E., Siontis, K. C., Pfeiffer, T., & Ioannidis, J. P. A. (2012). Perceived information gain from randomized trials correlates with publication in high–impact factor journals. *Journal of Clinical Epidemiology,**65*(12), 1274–1281. 10.1016/j.jclinepi.2012.06.009 22959593 10.1016/j.jclinepi.2012.06.009

[CR14] Funder, D. C., & Ozer, D. J. (2019). Evaluating effect size in psychological research: Sense and nonsense. *Advances in Methods and Practices in Psychological Science,**2*(2), 156–168. 10.1177/2515245919847202

[CR15] Gignac, G. E., & Szodorai, E. T. (2016). Effect size guidelines for individual differences researchers. *Personality and Individual Differences,**102*, 74–78. 10.1016/j.paid.2016.06.069

[CR16] Higgins, J. P. T., & Thompson, S. G. (2002). Quantifying heterogeneity in a meta-analysis. *Statistics in Medicine,**21*(11), 1539–1558. 10.1002/sim.1186 12111919 10.1002/sim.1186

[CR17] Huang, Y., Huang, X., Ebstein, R. P., & Yu, R. (2021). Intranasal oxytocin in the treatment of autism spectrum disorders: A multilevel meta-analysis. *Neuroscience and Biobehavioral Reviews,**122*, 18–27. 10.1016/j.neubiorev.2020.12.028 33400920 10.1016/j.neubiorev.2020.12.028

[CR18] Jurek, B., & Neumann, I. D. (2018). The oxytocin receptor: From intracellular signaling to behavior. *Physiological Reviews,**98*(3), 1805–1908. 10.1152/physrev.00031.2017 29897293 10.1152/physrev.00031.2017

[CR19] Kang, H., Glaser, B. D., Sartorius, A. M., Audunsdottir, K., Nærland, T., Andreassen, O. A., Westlye, L. T., & Quintana, D. (2022). *Effects of exogenous oxytocin administration on non-social executive function in humans: A preregistered systematic review and meta-analysis protocol*. OSF Preprints. 10.31219/osf.io/8fzdy 10.1038/s41380-024-02871-4 39827218

[CR20] Keech, B., Crowe, S., & Hocking, D. R. (2018). Intranasal oxytocin, social cognition and neurodevelopmental disorders: A meta-analysis. *Psychoneuroendocrinology,**87*, 9–19. 10.1016/j.psyneuen.2017.09.022 29032324 10.1016/j.psyneuen.2017.09.022

[CR21] Kvarven, A., Strømland, E., & Johannesson, M. (2020). Comparing meta-analyses and preregistered multiple-laboratory replication projects. *Nature Human Behaviour,**4*(4), Article 4. 10.1038/s41562-019-0787-z 10.1038/s41562-019-0787-z 31873200

[CR22] Lakens, D. (2013). Calculating and reporting effect sizes to facilitate cumulative science: A practical primer for t-tests and ANOVAs. *Frontiers in Psychology*, *4*. 10.3389/fpsyg.2013.00863 10.3389/fpsyg.2013.00863 PMC3840331 24324449

[CR23] Lakens, D. (2022). Sample size justification. *Collabra: Psychology,**8*(1), Article 33267. 10.1525/collabra.33267

[CR24] Lakens, D., & Evers, E. R. K. (2014). Sailing from the seas of chaos into the corridor of stability: Practical recommendations to increase the informational value of studies. *Perspectives on Psychological Science,**9*(3), 278–292. 10.1177/1745691614528520 26173264 10.1177/1745691614528520

[CR25] Lakens, D., Scheel, A. M., & Isager, P. M. (2018). Equivalence testing for psychological research: A tutorial. *Advances in Methods and Practices in Psychological Science,**1*(2), 259–269. 10.1177/2515245918770963

[CR26] Leppanen, J., Ng, K. W., Tchanturia, K., & Treasure, J. (2017). Meta-analysis of the effects of intranasal oxytocin on interpretation and expression of emotions. *Neuroscience and Biobehavioral Reviews,**78*, 125–144. 10.1016/j.neubiorev.2017.04.010 28467893 10.1016/j.neubiorev.2017.04.010

[CR27] Leppanen, J., Ng, K. W., Kim, Y.-R., Tchanturia, K., & Treasure, J. (2018). Meta-analytic review of the effects of a single dose of intranasal oxytocin on threat processing in humans. *Journal of Affective Disorders,**225*, 167–179. 10.1016/j.jad.2017.08.041 28837950 10.1016/j.jad.2017.08.041

[CR28] Leslie, M., Silva, P., Paloyelis, Y., Blevins, J., & Treasure, J. (2018). A systematic review and quantitative meta-analysis of the effects of oxytocin on feeding. *Journal of Neuroendocrinology,**30*(8), Article e12584. 10.1111/jne.12584 10.1111/jne.12584 PMC6292740 29480934

[CR29] Linden, A. H., & Hönekopp, J. (2021). Heterogeneity of research results: A new perspective from which to assess and promote progress in psychological science. *Perspectives on Psychological Science,**16*(2), 358–376. 10.1177/1745691620964193 33400613 10.1177/1745691620964193 PMC7961629

[CR30] Lovakov, A., & Agadullina, E. R. (2021). Empirically derived guidelines for effect size interpretation in social psychology. *European Journal of Social Psychology,**51*(3), 485–504. 10.1002/ejsp.2752

[CR31] McShane, B. B., Böckenholt, U., & Hansen, K. T. (2016). Adjusting for publication bias in meta-analysis: An evaluation of selection methods and some cautionary notes. *Perspectives on Psychological Science,**11*(5), 730–749. 10.1177/1745691616662243 27694467 10.1177/1745691616662243

[CR32] Nordahl-Hansen, A., Cogo-Moreira, H., Panjeh, S., & Quintana, D. (2022). *Redefining Effect Size Interpretations for Psychotherapy RCTs in Depression*. OSF Preprints. 10.31219/osf.io/erhmw 10.1016/j.jpsychires.2023.11.009 38000182

[CR33] Ooi, Y. P., Weng, S.-J., Kossowsky, J., Gerger, H., & Sung, M. (2017). Oxytocin and autism spectrum disorders: A systematic review and meta-analysis of randomized controlled trials. *Pharmacopsychiatry,**50*(01), 5–13. 10.1055/s-0042-109400 27574858 10.1055/s-0042-109400

[CR34] Page, M. J., Higgins, J. P., & Sterne, J. A. C., et al. (2024). Assessing risk of bias due to missing evidence in a meta-analysis. In J. P. Higgins, J. Thomas, J. Chandler, M. Cumpston, T. Li, & M. J. Page (Eds.), *Cochrane Handbook for Systematic Reviews of Interventions version 65. *Cochrane. Available from <Emphasis Type=&quot;Italic&quot;>cochrane.org/handbook</Emphasis>

[CR35] Panzarella, E., Beribisky, N., & Cribbie, R. A. (2021). Denouncing the use of field-specific effect size distributions to inform magnitude. *PeerJ,**9*, e11383. 10.7717/peerj.11383 34178435 10.7717/peerj.11383 PMC8210805

[CR36] Paterson, T. A., Harms, P. D., Steel, P., & Credé, M. (2016). An assessment of the magnitude of effect sizes: Evidence from 30 years of meta-analysis in management. *Journal of Leadership & Organizational Studies,**23*(1), 66–81. 10.1177/1548051815614321

[CR37] Peled-Avron, L., Abu-Akel, A., & Shamay-Tsoory, S. (2020). Exogenous effects of oxytocin in five psychiatric disorders: A systematic review, meta-analyses and a personalized approach through the lens of the social salience hypothesis. *Neuroscience and Biobehavioral Reviews,**114*, 70–95. 10.1016/j.neubiorev.2020.04.023 32348803 10.1016/j.neubiorev.2020.04.023

[CR38] Plonsky, L., & Oswald, F. L. (2014). How big is “big”? Interpreting effect sizes in L2 research. *Language Learning,**64*(4), 878–912. 10.1111/lang.12079

[CR39] Pogrow, S. (2019). How effect size (practical significance) misleads clinical practice: The case for switching to practical benefit to assess applied research findings. *The American Statistician,**73*(sup1), 223–234. 10.1080/00031305.2018.1549101

[CR40] Quintana, D. S. (2017). Statistical considerations for reporting and planning heart rate variability case–control studies. *Psychophysiology,**54*(3), 344–349. 10.1111/psyp.12798 27914167 10.1111/psyp.12798

[CR41] Quintana, D. S., & Guastella, A. J. (2020). An allostatic theory of oxytocin. *Trends in Cognitive Sciences,**24*(7), 515–528. 10.1016/j.tics.2020.03.008 32360118 10.1016/j.tics.2020.03.008

[CR42] R Core Team. (2023). *R: A Language and Environment for Statistical Computing*. R Foundation for Statistical Computing. https://www.R-project.org/

[CR43] Rochefort-Maranda, G. (2021). Inflated effect sizes and underpowered tests: How the severity measure of evidence is affected by the winner’s curse. *Philosophical Studies,**178*(1), 133–145. 10.1007/s11098-020-01424-z

[CR44] Rücker, G., Schwarzer, G., Carpenter, J. R., Binder, H., & Schumacher, M. (2011). Treatment-effect estimates adjusted for small-study effects via a limit meta-analysis. *Biostatistics,**12*(1), 122–142. 10.1093/biostatistics/kxq046 20656692 10.1093/biostatistics/kxq046

[CR45] Sabe, M., Zhao, N., Crippa, A., Strauss, G. P., & Kaiser, S. (2021). Intranasal oxytocin for negative symptoms of schizophrenia: Systematic review, meta-analysis, and dose-response meta-analysis of randomized controlled trials. *International Journal of Neuropsychopharmacology,**24*(8), 601–614. 10.1093/ijnp/pyab020 33890987 10.1093/ijnp/pyab020 PMC8378078

[CR46] Schäfer, T., & Schwarz, M. A. (2019). The meaningfulness of effect sizes in psychological research: Differences between sub-disciplines and the impact of potential biases. *Frontiers in Psychology*. 10.3389/fpsyg.2019.00813 31031679 10.3389/fpsyg.2019.00813 PMC6470248

[CR47] Schwarzer, G., Carpenter, J. R., & Rücker, G. (2015). *Meta-Analysis with R*. Springer International Publishing. 10.1007/978-3-319-21416-0

[CR48] Szucs, D., & Ioannidis, J. P. A. (2017). Empirical assessment of published effect sizes and power in the recent cognitive neuroscience and psychology literature. *PLoS Biology,**15*(3), Article e2000797. 10.1371/journal.pbio.2000797 28253258 10.1371/journal.pbio.2000797 PMC5333800

[CR49] Wang, Y., Wang, M.-J., Rong, Y., He, H.-Z., & Yang, C.-J. (2019). Oxytocin therapy for core symptoms in autism spectrum disorder: An updated meta-analysis of randomized controlled trials. *Research in Autism Spectrum Disorders,**64*, 63–75. 10.1016/j.rasd.2019.03.007

[CR50] Wickham, H., Hester, J., Chang, W., & Bryan, J. (2022). *devtools: Tools to Make Developing R Packages Easier*. https://devtools.r-lib.org/authors.html#citation

[CR51] Yang, X., Wang, W., Wang, X. T., & Wang, Y. W. (2021). A meta-analysis of hormone administration effects on cooperative behaviours: Oxytocin, vasopressin, and testosterone. *Neuroscience and Biobehavioral Reviews,**126*, 430–443. 10.1016/j.neubiorev.2021.03.033 33819546 10.1016/j.neubiorev.2021.03.033

[CR52] Zheng, W., Zhu, X.-M., Zhang, Q.-E., Yang, X.-H., Cai, D.-B., Li, L., Li, X.-B., Ng, C. H., Ungvari, G. S., Ning, Y.-P., & Xiang, Y.-T. (2019). Adjunctive intranasal oxytocin for schizophrenia: A meta-analysis of randomized, double-blind, placebo-controlled trials. *Schizophrenia Research,**206*, 13–20. 10.1016/j.schres.2018.12.007 30573406 10.1016/j.schres.2018.12.007

[CR53] Zhou, M. S., Nasir, M., Farhat, L. C., Kook, M., Artukoglu, B. B., & Bloch, M. H. (2021). Meta-analysis: Pharmacologic treatment of restricted and repetitive behaviors in autism spectrum disorders. *Journal of the American Academy of Child and Adolescent Psychiatry,**60*(1), 35–45. 10.1016/j.jaac.2020.03.007 32387445 10.1016/j.jaac.2020.03.007

