# Supplementary material for: A tutorial for calculating field-specific effect size distributions
Source: Behav Res Methods. 2026 Apr 29;58(6):146. doi: 10.3758/s13428-026-03003-2 (PMC13128698; doi:10.3758/s13428-026-03003-2)
Supplement: Supplementary file 1 — Supplementary file1 (DOCX 18 KB) [file 13428_2026_3003_MOESM1_ESM.docx]

# Loading package ---------------------------------

library(devtools)

devtools::install_github("berntgl/ESDist")

library(ESDist)

# Demonstrating package ---------------------------------

# esd_plot() =================================

# First, we will Plot the effect size distribution (ESD) and save it to a

# variable called 'plot1'

plot1 <- esd_plot(df = ot_dat,

es = yi,

es_type = "Hedges' g")

plot1

# If we want to calculate small/medium/large effect size benchmarks, we can do

# so by specifying a method. We can choose between the more common "quads"

# method and the "thirds" method that was used by Schäfer and Schwarz (2019).

# When we want to calculate such benchmarks, it is recommended to use absolute

# effect sizes only. Below, we create an ESD plot where we also highlight the

# benchmark estimates, as well as their 95% CIs by specifying "quads" as our

# `method` and by setting `ci` to TRUE. We also set `abs` to TRUE so we use only

# absolute effect sizes.

plot2 <- esd_plot(df = ot_dat,

es = yi,

es_type = "Hedges' g",

method = "quads",

ci = TRUE,

abs = TRUE)

plot2

# If we want to determine the range of effect sizes that a study design can

# detect, we can do so by setting a smallest effect size of interest (sesoi).

# In this case, we will pretend that we have a study that can detect an effect

# size of g = 0.3 with enough power. By adding the argument sesoi = 0.3, we can

# calculate and visualise the range of empirical effect sizes that we can

# reliably detect. In the code below, we create such a plot and save it to a

# variable called plot3. The plot also tells us that 55.17% of the empirical

# effect sizes found in this field are larger than 0.3. This means that with

# our study design, we can detect 55.17% of empirical effect sizes.

plot3 <- esd_plot(df = ot_dat, #we will now use absolute ES values only

es = yi,

es_type = "Hedges' g",

sesoi = 0.3,

abs = TRUE)

plot3

# Our dataset has several subgroups (e.g., healthy participants, ASD,

# Schizophrenia, etc.) that we might want to compare. In the code below we

# compare plots for each group and save the figure to a variable called plot4.

# Although, theoretically,we could compare all groups, some groups only have

# one or a few studies. As such, these groups are not very informative. The

# esd_plot() function therefore only includes groups with at least 20

# studies

plot4 <- esd_plot(df = ot_dat,

es = yi,

es_type = "Hedges' g",

grouping_var = group,

abs = TRUE)

plot4

# We can also use this type of visualisation to compare effect size benchmarks.

# We will use the absolute effect sizes for this and we will use the 'quads'

# approcah (note that it is also possible to use the thirds approach). We store

# the figure in a variable called plot5.

plot5 <- esd_plot(df = ot_dat,

es = yi,

es_type = "Hedges' g",

grouping_var = group,

method = "quads",

ci = TRUE,

abs = TRUE)

plot5

# esd_plot_pba() =================================

# Unfortunately, a lot of published empirical results are prone to biases, such

# as publication bias. The metasens package (Schwarzer et al., 2023) uses

# limit meta-analysis to adjust individual effect sizes for publication bias.

# The esd_plot_pba() function uses the limitmeta function from metasens to plot

# the distribution of adjusted effect sizes against the distribution of

# unadjusted effect sizes. We only need to add one new argument, "se", which

# corresponds to the column with standard error.

plot6 <- esd_plot_pba(df = ot_dat,

es = yi,

se = sei,

es_type = "Hedges' g")

plot6

# We can also visualise the adjusted effect size benchmarks for our

# distribution, by setting the method argument. We save the result to plot7.

plot7 <- esd_plot_pba(df = ot_dat,

es = yi,

se = sei,

es_type = "Hedges' g",

method = "quads",

ci = TRUE,

abs = TRUE)

plot7

# Finally, we can visualise the range of detectable ESs based on a sesoi.

plot8 <- esd_plot_pba(df = ot_dat,

es = yi,

se = sei,

es_type = "Hedges' g",

sesoi = 0.3,

abs = TRUE)

plot8

# esd_table() =================================

# The esd_table() function allows you to calculate effect size benchmarks. In

# its simplest form, it takes a dataframe and the name of the column housing

# all absolute effect sizes.

table1 <- esd_table(df = ot_dat,

es = yi)

table1

# We can check the skewness of our distribution by setting `bowley` to TRUE.

table2 <- esd_table(df = ot_dat,

es = yi,

bowley = TRUE)

table2

# In case we want to compare benchmarks between groups, we can define our

# grouping variable in the function as well. In this case, we also get a summary

# of all effect sizes in the bottom row. We save the results to a variable

# called table2a. Again, we will calculate benchmarks based on the absolute ESD.

table3 <- esd_table(df = ot_dat,

es = yi,

grouping_var = group,

abs = TRUE)

table3

# We can also calculate the 95% CI around the benchmark estimates using

# bootstrapping by setting `ci` to TRUE.

table4 <- esd_table(df = ot_dat,

es = yi,

ci = TRUE,

abs = TRUE)

table4

# We can save this table as a .csv file by adding another argument to our

# function. By setting csv_write = TRUE, and by specifying a file_name argument,

# we save our table as a .csv file to the directory we specify in our R

# environment. Below, we copied the definition of table3, but added the command

# to save the table as a .csv file.

esd_table(df = ot_dat,

es = yi,

grouping_var = group,

abs = TRUE,

csv_write = TRUE,

path_file_name = "/path/to/file.csv")

# esd_table_pba() =================================

# We can also calculate benchmarks for the publication bias-adjusted ESD. Like

# the plotting function, the esd_table_pba() function takes a 'limitmeta'

# object. Here, we use the same l1 object we used earlier.

table5 <- esd_table_pba(df = ot_dat,

es = yi,

se = sei,

abs = TRUE)

table5

# In case you want to create benchmarks (and adjusted benchmarks) per group,

# we simply need to specify the `grouping_var` again.

table6 <- esd_table_pba(df = ot_dat,

es = yi,

se = sei,

grouping_var = group,

abs = TRUE)

table6

# Finally, we can also calculate the 95% CIs around the pba benchmark estimates

# by setting `ci` to TRUE.

table7 <- esd_table_pba(df = ot_dat,

es = yi,

se = sei,

ci = TRUE,

abs = TRUE)

table7

# esd_perc() =================================

# If we want to very simply calculate the percentile of a specific value, we

# can use the esd_perc() function. In the code below we calculate the percentile

# corresponding to g = 0.2. Note that we use yi_abs, as we are only interested

# in the size of all effects, not in their direction.

esd_perc(df = ot_dat,

es = yi,

value = 0.2)
