# Supplementary material for: A tutorial for calculating field-specific effect size distributions
Source: Behav Res Methods. 2026 Apr 29;58(6):146. doi: 10.3758/s13428-026-03003-2 (PMC13128698; doi:10.3758/s13428-026-03003-2)
Supplement: Supplementary file 2 — Supplementary file2 (DOCX 15 KB) [file 13428_2026_3003_MOESM2_ESM.docx]

# Loading and filtering data =================================

library(dplyr)

library(ESDist)

# The ESDist package includes two data files; ot_dat_raw, which contains all

# effect sizes from the included meta-analyses, and ot_dat, which is the

# filtered version of that data. The code below demonstrates how the data can

# be filtered using the ci_to_se() helper function.

#load data

dat <- ot_dat_raw

# We create a new dataset called ot_dat, which we will filter.

ot_dat <- dat

# We will convert some effect sizes to Hedges' g, so we create a new column

# with effect sizes as they were reported.

ot_dat$raw_es <- ot_dat$yi

ot_dat$raw_es[ot_dat$favours_oxytocin == "negative"] <- -1 * ot_dat$raw_es[ot_dat$favours_oxytocin == "negative"]

# We also give every single effect size an ID, so we know which ones are

# eventually filtered out.

ot_dat$ID <- seq.int(nrow(ot_dat))

# We calculate Standard error for each effect size from the 95% CI.

ot_dat$sei[is.na(ot_dat$sei)] <- ci_to_se(ot_dat$lower[is.na(ot_dat$sei)], ot_dat$upper[is.na(ot_dat$sei)])

ot_dat$raw_se <- ot_dat$sei

# Next, we convert all effect sizes we can to Hedges' g, based on group sizes.

# We use the simplified unbias factor given by Hedges (1981), based on the

# degrees of freedom, which are calculated differently based on study design.

# First we calculate the df for each effect size and add them to a df column.

# Next, we calculate all effect sizes based on Hedges' factor and round

# each effect size to two decimal places.

ot_dat$df[ot_dat$design == 'Between'] <- ot_dat$n1[ot_dat$design == 'Between'] + ot_dat$n2[ot_dat$design == 'Between'] - 2

ot_dat$df[ot_dat$design == 'Within'] <- ot_dat$n_total[ot_dat$design == 'Within'] - 1

ot_dat$h_factor <- (1 - (3/((4*ot_dat$df)-1)))

ot_dat$yi[ot_dat$es_type != "Hedges' g"] <- ot_dat$raw_es[ot_dat$es_type != "Hedges' g"] * ot_dat$h_factor[ot_dat$es_type != "Hedges' g"]

ot_dat$sei[ot_dat$es_type != "Hedges' g"] <- sqrt((ot_dat$raw_se[ot_dat$es_type != "Hedges' g"]^2) * (ot_dat$h_factor[ot_dat$es_type != "Hedges' g"]^2))

ot_dat$yi <- round(ot_dat$yi, digits = 3)

ot_dat$sei <- round(ot_dat$sei, digits = 3)

ot_dat$yi_abs <- abs(ot_dat$yi)

# Filter out the effect sizes with lowest SE per group per study (some studies

# have multiple groups), and the effects with the lowest SE. In case some

# effects from the same study have the same SE, we only use the effect size

# that is closest to zero in absolute terms.

ot_dat <- ot_dat %>%

group_by(study_doi, group) %>%

filter(!is.na(yi)) %>%

filter(sei == min(sei)) %>%

filter(abs(yi) == min(abs(yi))) %>%

ungroup()

# The filtering process messes up the data type a bit, so let's turn it back

# into a dataframe

ot_dat <- as.data.frame(ot_dat)
