# Supplementary material for: A tutorial for calculating field-specific effect size distributions
Source: Behav Res Methods. 2026 Apr 29;58(6):146. doi: 10.3758/s13428-026-03003-2 (PMC13128698; doi:10.3758/s13428-026-03003-2)
Supplement: Supplementary file 3 — Supplementary file3 (DOCX 15 KB) [file 13428_2026_3003_MOESM3_ESM.docx]

# install.packages(c("metafor", "meta", "weightr"))

library(metafor)

library(meta)

library(weightr)

################################

# Random-effects meta-analysis #

################################

re <- rma(yi = yi,

sei = sei,

data = ot_dat,

method = "REML")

print(re)

# Mean estimate is .189, se = .045.

#################

# Trim and Fill #

#################

# Funnel plot

metafor::funnel(re, main = "Random effects")

# Trim and fill using metafor

res.tf <- trimfill(re)

print(res.tf)

# Conclusion: Random effects model and trim and fill are the exact same - no

# effects were imputed - no evidence of publication bias.

# Funnel plot with imputed studies

metafor::funnel(res.tf, main = "Trim and fill")

##########################

# Weight-selection model #

##########################

# Define cutpoints for p-value selection model.

cut_points <- c(.25, .5)

wfit <- weightfunct(effect = ot_dat$yi,

v = ot_dat$sei^2,

steps = cut_points,

table = TRUE)

print(wfit)

# Considerable evience for heterogeneity, and considerable evidence for

# publication bias. Although the adjusted estimate (.177) is not much different

# from the original estimate (.188), the adjusted SE (.097) is more than double

# that of the original SE (.044).

#######################

# Limit meta-analysis #

#######################

# Create meta object

meta <- metagen(TE = yi,

seTE = sei,

data = ot_dat,

sm = "SMD",

method.tau = "REML",

common = FALSE,

random = TRUE)

# Limit meta-analysis (adjusts for small-study effects)

limit <- limitmeta(meta)

print(limit)

# Considerable evidence for heterogeneity (Q = 902.75, df = 181, p < .001, df).

# Considerable evidence for publication bias (Q-Q' = 26.24, df = 1, p-value < .001),

# with the adjusted estimate (g = .104, CI = [-.036; 0.245]) differing

# substantially from the original estimate (g = .189, CI = [.101; .276])

########################################

# Interpretation guide #

########################################

# - Trim and fill:

# If the trim-and-fill pooled estimate differs meaningfully from the original,

# and many studies are imputed on one side of the funnel, this suggests

# possible publication bias / small-study effects.

#

# - Weight-selection model:

# `adj_est` is an estimate corrected for selective publication.

# A large difference from the unadjusted meta-analytic estimate suggests bias.

#

# - Limit meta-analysis:

# The `TE.adjust` is an adjusted estimate accounting for small-study effects.

# A notable shift from TE.random suggests possible bias.
