# Supplementary material for: A tutorial for calculating field-specific effect size distributions
Source: Behav Res Methods. 2026 Apr 29;58(6):146. doi: 10.3758/s13428-026-03003-2 (PMC13128698; doi:10.3758/s13428-026-03003-2)
Supplement: Supplementary file 4 — Supplementary file4 (PDF 104 KB) [file 13428_2026_3003_MOESM4_ESM.pdf]

Table S1

A table containing the effect size benchmarks as shown in Figure 1A

Study	Field	Small	Medium	Large	*K*
Lovakov & Agadullina (2021)	Social psychology	0.15	0.36	0.65	6447
Quintana (2017)	Heart rate variability	0.26	0.51	0.88	297
Nordahl-Hansen et al. (2023)*	Psychotherapy RCTs in depression	0.27	0.53	0.86	366
Cherubini & MacDonald (2021	Human endothelial function research	0.28	0.69	1.21	752
Plonsky & Oswald	Second language research	0.45	0.71	1.08	236
Szucs & Ioannidis (2017) §	Medical science	0.23		0.91	348
Szucs & Ioannidis (2017) §	Psychology	0.29		0.96	2261
Szucs & Ioannidis (2017) §	Neuroscience	0.34		1.22	1192

Note. Table listing the small, medium, and large effect size benchmarks and number of studies (K) included in the calculation of those benchmarks, for different scientific fields. The benchmarks for all studies are based on the 25th, 50th, and 75th percentiles. Studies with a section sign (§) did not report a 50th percentile effect size. The benchmarks from studies with an asterisk (*) are adjusted for publication bias. Studies with a section sign (§) did not report a median value. RCT = Randomised controlled trial.

Table S2

A table containing the effect size benchmarks as shown in Figure 1B

Study	Field	Small	Medium	Large	*K*
Schäfer & Schwarz (2019)*	Psychology	0.04	0.16	0.41	89
Bosco et al. (2014)	Applied psychology	0.07	0.16	0.29	147 328
Gignac & Szodorai (2016)	Individual differences research	0.11	0.19	0.29	708
Brydges (2019)	Gerontology	0.12	0.20	0.32	1108
Paterson et al. (2017)	Management studies	0.12	0.20	0.31	686
Lovakov & Agadullina (2021)	Social psychology	0.12	0.24	0.41	12170
Schäfer & Schwarz (2019)	Psychology	0.20	0.36	0.62	684
Plonsky & Oswald	Second language research	0.25	0.37	0.54	175

Note. Table listing the small, medium, and large effect size benchmarks and number of studies (K) included in the calculation of those benchmarks, for different scientific fields. The benchmarks for all studies are based on the 25th, 50th, and 75th percentiles, except for the studies by Schäfer and Schwarz, which are based on the 16.65th, 50th, and 83.35th percentiles. The benchmarks from studies with an asterisk (*) have been adjusted for publication bias.
